# Chromosome-level draft genome sequences of three isolates of the toxigenic fungus *Claviceps purpurea* showing structural rearrangements

**DOI:** 10.1128/MRA.00234-23

**Published:** 2023-09-21

**Authors:** Thomas E. Witte, Carmen Hicks, Parivash Shoukouhi, Kasia Dadej, Wendy Findlay, Miao Liu, David P. Overy

**Affiliations:** 1 Ottawa Research and Development Centre, Agriculture & Agri-Food Canada, Ottawa, Ontario, Canada; University of California, Riverside, California, USA

**Keywords:** Clavicepitaceae, ergot, whole genome, nanopore, *Claviceps purpurea*, mycology, plant pathogens, mycotoxins

## Abstract

The whole genomes of three *Claviceps purpurea* strains were sequenced using Oxford Nanopore Technologies’ MinION and assembled into complete, chromosome-level assemblies. The *C. purpurea* genome consists of eight conserved chromosomes, with evidence of inter-chromosomal structural rearrangements between strains.

## ANNOUNCEMENT

*Claviceps purpurea* causes ergot disease in cereal crops and is of particular concern due to its production of potent neurotoxic ergot alkaloids. In a previous study (1), three *C. purpurea* isolates (LM04, LM60, and LM72), obtained from infected triticale (*x Triticosecale*), oat (*Avena sativa*), and black grass (*Alopecurus myosuroides*), exhibited variations in ergot alkaloid production and associated biosynthetic gene cluster sequences.

LM04, LM60, and LM72 were cultured on potato dextrose agar (PDA) for 10 days. Mycelia were grounded in liquid N₂, and gDNA was extracted using a cetyltrimethylammonium bromide (CTAB) protocol (2), evaluated for integrity/purity using TapeStation Genomic DNA ScreenTapes (Agilent) and DropSense 16 (Trinean), and quantified using a Qubit 2.0 Fluorometer (Invitrogen by Life Technologies). DNA libraries were prepared using 1 µg of gDNA following the Ligation SQK-LSK-109 protocol (Oxford Nanopore Technologies), and an average of 25 fmol were loaded onto a FLO-MIN106 flow cell and run for 48 h on a MinION Mk1b device, with Fast basecalling enabled, read filtering min-qscore = 7, using applications MinKNOW 20.06.5, MinKNOW Core 4.0.5, Bream 6.0.10, and Guppy 4.0.9. The number of raw reads and other statistical parameters are listed in Table 1.

**TABLE 1:** Host, provenance, and genome statistics for three *C. purpurea* genomes

Strain name	LM04	LM60	LM72	*C. purpurea* 20.1^*a*^
Host	*Triticale*	*Avena sativa*	*Alopecurus myosuroides*	*Secale cereale*
Origin	Canada, Manitoba	Canada, Manitoba	UK, Cambridge	Germany, Hohenheim
Bioproject	PRJNA901936	PRJNA901936	PRJNA901936	N/A
Biosample accession	SAMN31741817	SAMN31741818	SAMN31741819	N/A
Genome accession	GCA_029405315.1	GCA_029405515.1	GCA_029405325.1	GCA_000347355.1
No. of chromosomes	8	8	8	191 (scaffolds)
Total length (bases)	33,155,907	33,433,899	34,178,275	32,091,443
N50 (assembly, bases)	4,292,713	4,364,879	4,441,790	433,200
N50 (reads, bases)	9,365	2,881	4,422	N/A
GC (%)	51.7	51.7	51.7	51.5
SRA accession	SRR22300868	SRR22300867	SRR22300866	N/A
No. of reads (Nanopore)	1,554,652	5,861,673	1,748,000	N/A
Total read length (bases)	8,219,792,840	12,082,634,056	3,082,852,418	N/A
Genome coverage (est. for 34 Mb genome)	242X	355X	91X	N/A

^*a*^ published in Schardl et al. (3), included here for comparison.

Genomes were assembled using two Nanopore long read assemblers, CANU v1.8 (4) and NECAT v0.0.1 (5), with default settings and an estimated genome size of 36 Mb. The resulting assemblies were polished using Nanopolish v. 0.13.2 (6), followed by two rounds of Pilon v1.23 (7) to correct nanopolished assemblies using Illumina reads mapped to the assembly through BWA v0.7.17 (8). The Illumina NextSeq sequences for the three strains are publicly available (9, 10). Genome metrics were obtained using QUAST v5.0.2 (11). There were no structural disagreements between the two assembler outputs; however, NECAT was more successful in completing the telomeric regions of isolates LM60 and LM04, identified by detection of canonical TTAGGG repeats, and was therefore used to produce the final assemblies. Telomeres were predicted by manual comparison of contig ends in Geneious.

**Fig 1:**
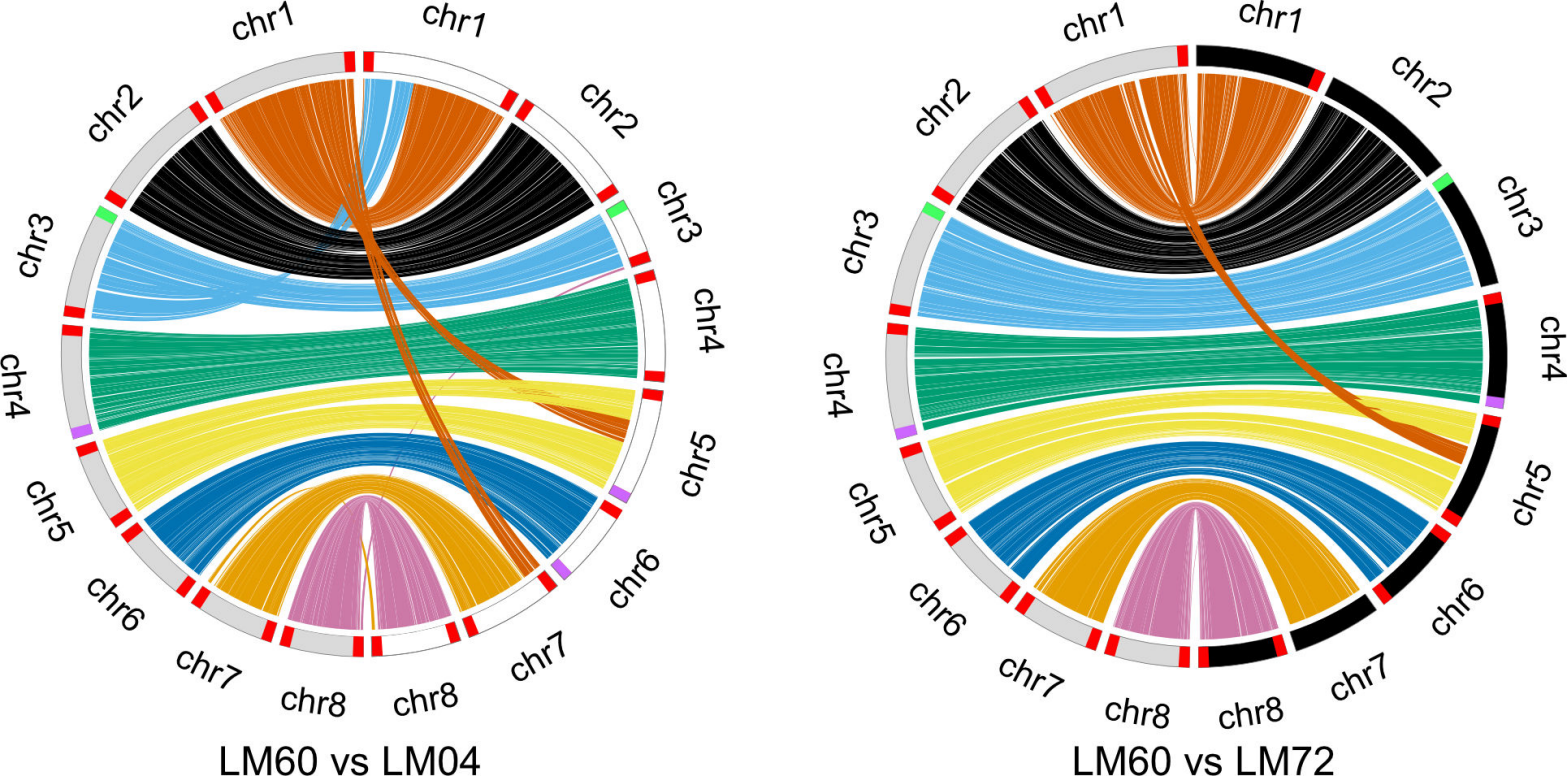
Syntenic comparison of three *C. purpurea* genomes: LM60 (gray chromosomes), LM04 (white chromosomes), and LM72 (black chromosomes). Telomeres are labeled based on detection of canonical telomeric “TTAGGG” repeats (red), rDNA repeats (green), and noncanonical repeats (purple). Ribbons connecting chromosomes represent syntenic regions, colored by association with LM60 chromosomes. Plots are created using circos v0.69–8 (12).

The *C. purpurea* whole genome assemblies each consist of eight chromosomes ranging in size from under ~1 Mb to ~6 Mb, for a total of between 33.2 and 34.2 Mb (Table 1). Synteny comparisons of LM04 and LM60 indicate they are complete, whereas LM72 lacks telomeric repeats (Fig. 1). Chromosomes were named based on their sizes in the LM60 assembly. Some chromosomes appear to terminate in noncanonical repeats, with each repeat consisting of 674 base pairs with a relatively low GC content (43.1%). These assemblies improve upon the current best quality publicly available *C. purpurea* assembly (Table 1) (3).

Notably, chromosomal structural rearrangements exist between isolates (Fig. 1). Assemblies were checked for errors by aligning long reads to the assemblies using Geneious v2022.2.2 and manually verifying continuous coverage at the putative rearrangement sites, with continuous coverage detected at all sites.

## Data Availability

All sequences are deposited in GenBank under the bioproject PRJNA901936 with the following accession numbers: LM04, JARFYB000000000; LM60, JARFYA000000000; and LM72, JARFXZ000000000. The versions described in this paper are the first versions (JARFYB010000000, JARFYA010000000, and JARFXZ010000000). Raw reads are deposited in Sequence Read Archive with the following accession numbers: LM04, SRR22300868; LM60, SRR22300867; and LM72, SRR22300866.
